# Using Routine Hemoglobin A1c Testing to Determine the Glycemic Status in Psychiatric Inpatients

**DOI:** 10.3389/fendo.2017.00053

**Published:** 2017-03-28

**Authors:** Pratyusha Naidu, Leonid Churilov, Alvin Kong, Richard Kanaan, Henry Wong, Arielle Van Mourik, Anthony Yao, Elizabeth Cornish, Mariam Hachem, Graeme K. Hart, Elizabeth Owen-Jones, Raymond Robbins, Que Lam, Katherine Samaras, Jeffrey D. Zajac, Elif I. Ekinci

**Affiliations:** ^1^Department of Endocrinology, Austin Health, Heidelberg, VIC, Australia; ^2^The Florey Institute of Neuroscience and Mental Health, Heidelberg, VIC, Australia; ^3^Department of Psychiatry, Austin Health, Heidelberg, VIC, Australia; ^4^Department of General Medicine, Austin Health, Heidelberg, VIC, Australia; ^5^University of Melbourne (Austin Health), Parkville, VIC, Australia; ^6^Department of Intensive Care, Austin Health, Heidelberg, VIC, Australia; ^7^Austin Centre for Applied Clinical Informatics, Austin Health, Heidelberg, VIC, Australia; ^8^Department of Administrative Informatics, Austin Hospital, Heidelberg, VIC, Australia; ^9^Department of Pathology, Austin Hospital, Heidelberg, VIC, Australia; ^10^Department of Endocrinology, St Vincent’s Hospital, Sydney, NSW, Australia; ^11^Faculty of Medicine, St Vincent’s Clinical School, UNSW, Sydney, NSW, Australia; ^12^Menzies School of Health Research, Darwin, VIC, Australia

**Keywords:** diabetes, pre-diabetes, psychiatry inpatients, atypical antipsychotics, obesity

## Abstract

**Aim:**

Using routine hemoglobin A1c (HbA1c) testing to describe the prevalence, characteristics, and length of stay (LOS) of psychiatry inpatients with type 2 diabetes compared to those with pre-diabetes and those without diabetes.

**Methods:**

In this prospective observational study, all inpatients aged greater than 30 years admitted to the Austin Health Psychiatry Unit, a major tertiary hospital, affiliated with the University of Melbourne, between February 2014 and April 2015 had routine HbA1c testing as part of the Diabetes Discovery Initiative. Patients were divided into three groups: diabetes (HbA1c ≥ 6.5%, 48 mmol/mol), pre-diabetes (HbA1c 5.7–6.4%, 39–46 mmol/mol), or no diabetes (HbA1c ≤ 5.6%, 38 mmol/mol). Baseline characteristics, co-morbidities, psychiatric illnesses, and treatment were recorded.

**Results:**

There were a total of 335 psychiatry inpatients (median age 41 years). The most prevalent diagnoses were schizophrenia, depression, and substance abuse. Of the 335 psychiatric inpatients, 14% (*n* = 46) had diabetes and 19% (*n* = 63) had pre-diabetes, a prevalence threefold greater than in the aged matched general population. Compared to inpatients with pre-diabetes and no diabetes, those with diabetes were older and were at least twice as likely to have hypertension, obesity, and hyperlipidemia (all *p* ≤ 0.002). In multivariable analyses, diabetes was associated with increasing age (*p* = 0.02), substance abuse (*p* = 0.04), dyslipidaemia (*p* = 0.03), and aripiprazole use (*p* = 0.01). Patients with diabetes also had a 70% longer expected LOS (95% CI: 20–130%; *p* = 0.001), compared to those with pre-diabetes and no diabetes.

**Conclusion:**

Despite relative youth, one-third of all psychiatric inpatients above the age of 30 have diabetes or pre-diabetes. Presence of diabetes in psychiatric inpatients is associated with older age, substance abuse, and longer LOS. Routine inpatient HbA1c testing provides an opportunity for early detection and optimization of diabetes care.

## Introduction

Diabetes and mental illness are major causes of disability and mortality and consume a significant aspect of the Australian health budget (1, 2). The age-standardized prevalence of type 2 diabetes in Australia has doubled over the last decade according to the Australian Institute of Health and Welfare Data, with 4.2% of population having diabetes between 2011 and 2012 compared to 1.5% in 1989–90 (3). We recently showed that approximately one-third of general inpatients aged >54 years admitted to our hospital had type 2 diabetes (4). A recent Melbourne Public Hospitals Diabetes Inpatient Audit revealed the prevalence of diabetes ranged from 16 to 35% in different hospitals across the greater metropolitan region (5).

Likewise, the prevalence and effects of mental illness are many-fold. Many Australians experience mental illness at some point in their lives (6). In a large Australian survey of approximately 1,800 community-dwelling people with psychotic illness, approximately 20% had diabetes (6). This is a concern, given the relative youth of people affected by both diabetes and psychosis (~88% were aged less than 55 years) and that the age-matched prevalence of type 2 diabetes in the community at 55 years was only 8% (3). The survey examined patients with psychotic disorders in the community, and there are limited data in the inpatient setting. Furthermore, the survey did not delineate additional clinical characteristics in the cohort of patients with diabetes (6). There are no Australian data on the prevalence of diabetes and pre-diabetes in psychiatry inpatients.

Psychiatry inpatients are likely to be more vulnerable to diabetes, largely because of the acuity of the mental illness as well as the likelihood they will be exposed to higher doses or multiple medications that increase diabetes risk (6).

Diabetes and mental illness share a bidirectional interface where both influence each other (7, 8). This is important to appreciate when trying to understand the underlying pathophysiology and characteristics of patients who might be at increased risk. Many studies have shown a link between antipsychotic medication and impaired glycemia. A systematic review and meta-analysis undertaken in 2008 examined 11 studies to date (9). It demonstrated that the relative risk of diabetes in patients with schizophrenia prescribed second-generation versus first-generation antipsychotics was 1.32 (95% CI 1.15–1.51) (9). However, this observation is likely to be biased due to the methodological limitations in many of the studies included (9).

Measuring hemoglobin A1c (HbA1c) as a means of identifying patients with diabetes is now recommended by the American Diabetes Association (ADA), as it is considered to be a more stable parameter, less affected by factors, such as fasting status, glucocorticoid use, or stress hyperglycemia (10, 11). In a small study of people with mental illness treated with antipsychotic medications in the community, HbA1c measurements detected a greater number of cases with pre- diabetes or diabetes, compared with the use of the fasting blood glucose criteria (12). Little has been documented about the use of HbA1c in the inpatient population with severe mental illness.

Using routine HbA1c testing, the aim of this study was, therefore, to determine the prevalence, characteristics, and length of stay (LOS) of psychiatry inpatients with diabetes compared to those with pre-diabetes or without diabetes.

## Materials and Methods

All patients admitted to the inpatient psychiatry unit at Austin Health, a major tertiary hospital, affiliated with the University of Melbourne, during the study period (February 2014 to April 2015) above the age of 30 were included in this prospective observational study. During the 14-month study period, all inpatients underwent HbA1c testing on admission *via* Cerner Millennium Information System Program, as part of routine clinical care as part of the Diabetes Discovery Initiative (4). Cerner Millennium Information System Program is the electronic health system used at Austin Health. We wrote a “discern rule” to identify all inpatient admissions with the following eligibility criteria: age ≥ 30 years, acute psychiatric unit admission, and no HbA1c recorded within 3 months. If these criteria were satisfied, an automatic request for HbA1c was generated for routine blood collection. All HbA1c results were reported *via* Cerner and were accessible to the patients’ treating clinicians. As part of the Diabetes Discovery Initiative, all inpatients with HbA1c ≥ 8.5% (69 mmol/mol) were automatically reviewed by the endocrinology team to intensify diabetes treatment, screen for complications, and optimize management (4). A paragraph describing the HbA1c result and its interpretation was automatically inserted into each discharge summary to patients’ local doctors (4).

Baseline characteristics, including demographic data such as age, sex, residence, marital status, employment, drug, and alcohol history were collected. Information regarding comorbidities was obtained from each patient’s medical record to calculate the Charlson Index score without diabetes. This validated method of weighting the impact of chronic disease, assigns each chronic condition a score of 1, 2, 3, or 6, depending on severity and impact on mortality (4). Specific comorbidities, including diabetes, hypertension, obesity (using weight and body mass index), and dyslipidemia, were recorded. If the patient had diabetes, information was collected on diabetes duration and treatment.

Data on the diagnosis of psychiatric illnesses were collected using the following classification: schizophrenia, schizoaffective disorder, bipolar affective disorder, depression, other psychosis, substance abuse, eating disorders, and other diagnoses. Duration of illness and treatment data were documented. Patients with two or more psychiatric diagnoses were noted. Psychotropic medications were categorized as anti-depressants, mood stabilizers, typical or atypical anti-psychotics, and both. Antipsychotic medication type was recorded.

Biochemical laboratory values (HbA1c, haemoglobin, creatinine, estimated glomerular filtration rate, and fasting lipid profile) were extracted from medical records within three months of the admission date. The length of inpatient stay was recorded.

For patients who had multiple admissions, only the data from the initial admission was included in this study. This study was carried out in accordance with the recommendations of Austin Health Research Ethics Committee (LNR/15/Austin/41), who waived the need for informed consent for a planned practice change agreed to by the hospital senior medical staff members as part of the Austin Health Diabetes Discovery Initiative. The protocol was approved by the Austin Health Research Ethics Committee.

Patients were categorized into three glycemic categories according to the ADA guidelines using HbA1c definitions as follows: “No diabetes” defined as HbA1c less than or equal to 5.6% (38 mmol/mol), “Pre-diabetes” defined as HbA1c greater than or equal to 5.7% and less than or equal to 6.4% (39–46 mmol/mol), and “Diabetes” defined as HbA1c greater than or equal to 6.5% (48 mmol/mol) (13).

### Statistical Methods

Glycemic categories were compared for baseline characteristics, psychiatric diagnosis, duration of psychiatric illness, psychotropic medication type, and LOS. Continuous explanatory variables were summarized as medians with interquartile ranges (IQRs) and compared with the use of Wilcoxon tests or Kruskal–Wallis tests as appropriate and categorical explanatory variables were reported as percentages and compared with χ^2^ tests or Fisher’s exact test, as appropriate. Multivariable analyses were based on negative binomial regression modeling for the LOS calculations (LOS expressed as a count of days) and on logistic regression modeling for binary outcomes. When investigating the association between the presence of diabetes and other clinical factors, the statistically significant variables from the univariate analysis were included in a multivariable analysis. Standard diagnostics of model fit and collinearity (based on mean variance inflating factors and condition numbers) were conducted. All *p* values were calculated from two-tailed tests of statistical significance and *p* < 0.05 was regarded as statistically significance. All analyses were performed with Stata software V.13.0 (StataCorp, College Station, TX, USA).

## Results

A total of 335 psychiatry inpatients formed the study cohort. Fourteen percent of these patients (95% CI: 10–18%, *n* = 46) had diabetes, 19% (95% CI: 15–23%, *n* = 63) had pre-diabetes, and 67% (95% CI: 62–72%, *n* = 226) had no diabetes. Only two patients had new diagnosis of diabetes, and there were none with type 1 diabetes. The median HbA1c was 7.3% (IQR 6.2, 8.7) or 56 mmol/mol (IQR 44, 72) in the diabetes group, 5.8% (IQR 5.7–5.9) or 40 mmol/mol (IQR 39–41) in the pre-diabetes group, and 5.4% (IQR 5.2–5.5) or 36 mmol/mol (IQR 33–37) in the no diabetes group (Table 1).

**Table 1:** **Patient characteristics by diabetes status**.

Clinical characteristics	*N*	Diabetes	Pre-diabetes	No diabetes	*p*-value*
Number (%)	335	46 (13.7%)	63 (18.8%)	226 (67.5%)	
Gender, male (%)	335	32 (70%)	40 (64%)	123 (54%)	0.106
Unemployed (%)	335	41 (89%)	53 (84%)	164 (73%)	0.017
Median (IQR) age (years)	335	49 (39–62)	46 (36–55)	40 (34–48)	<0.001
Median (IQR) (HbA1c) (%)	335	7.3 (6.2–8.7)	5.8 (5.7–5.9)	5.4 (5.2–5.5)	<0.001
Median (IQR) HbA1c (mmol/mol)	335	56 (44–72)	40 (39–41)	36 (33–37)	<0.001
Median (IQR) duration of psychiatric illness (years)	335	12 (6–18)	10 (4–19)	9 (3–18)	0.215
Median length of stay (IQR) (days)	335	29 (14–81)	21 (9–26)	17 (9–31)	0.003
Charlson Index of co-morbidities (IQR) (score)	335	1 (1–2)	0 (0–1)	0 (0–0)	<0.001
Hypertension (%)	335	10 (22%)	9 (14%)	14 (6%)	0.002
Hyperlipidemia (%)	335	13 (28%)	6 (10%)	14 (6%)	<0.001
Obesity (%)	301	20 (44%)	15 (23%)	38 (17%)	0.001

Continuous explanatory variables were summarized as medians with interquartile ranges (IQRs) and compared with the use of Kruskal–Wallis tests and categorical explanatory variables were reported as percentages and compared with χ^2^ tests. All *p* values for three group comparisons were calculated from two-tailed tests of statistical significance and *p < 0.05 was regarded as statistically significant.

### Demographics

Compared to patients with pre-diabetes and no diabetes, patients with diabetes were predominantly older (median, IQR): age 49 (39–62) versus those with pre-diabetes (age 46, IQR 36–55) and no diabetes (age 40, IQR 34–48) (*p* = < 0.001) (Table 1).

**Figure 1:**
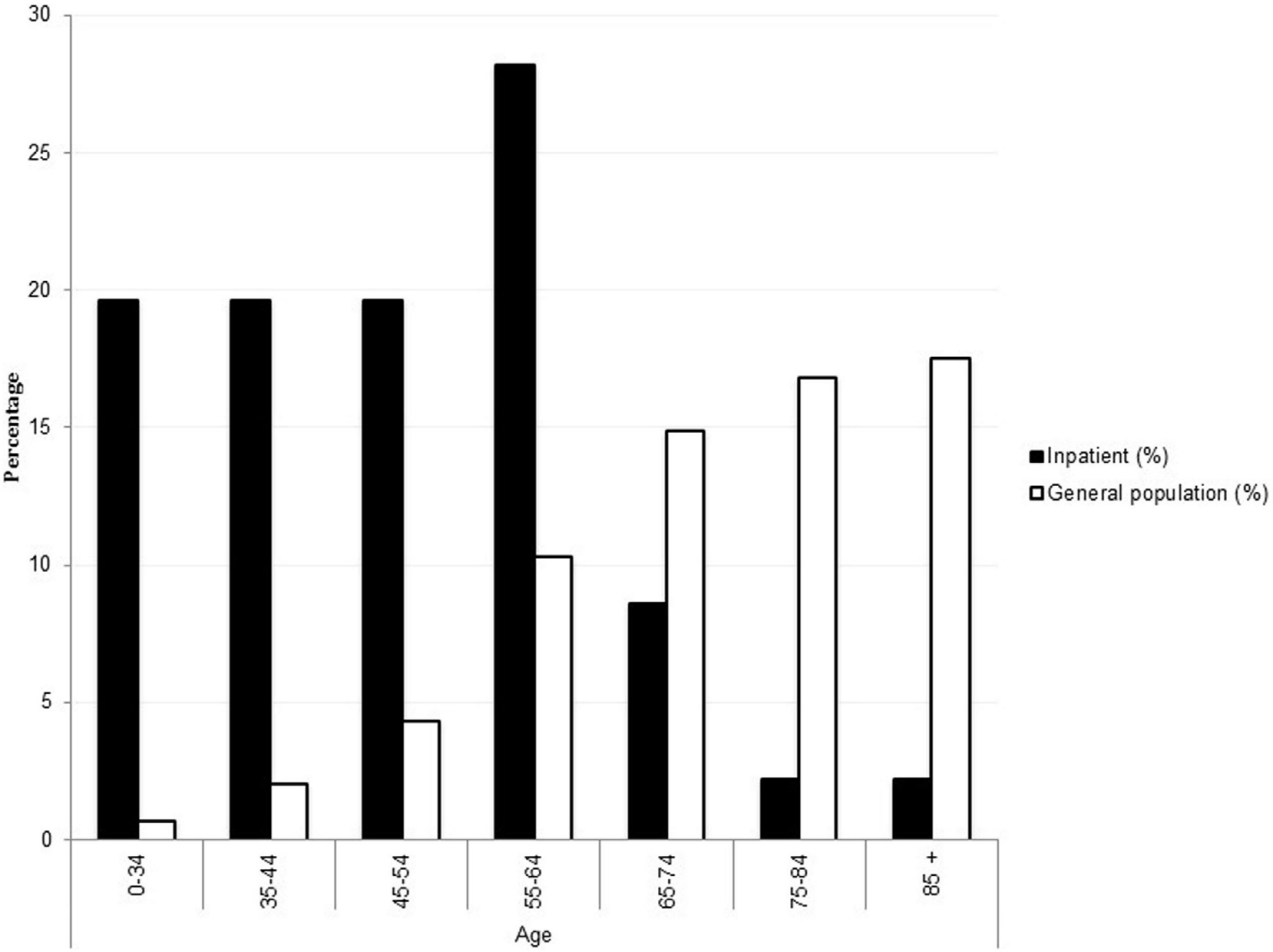
**Percentage of adults with diagnosed diabetes by age group in the inpatient psychiatry population versus general age-matched population with diabetes**. General age-matched population data are adapted from Australian Bureau of Statistics (14) and reproduced without permission.

The proportion of psychiatry inpatients with diabetes by age group was disproportionately higher and skewed in the opposite direction toward younger age, compared to community diabetes prevalence (Figure 1) (14).

Patients with diabetes compared to those with pre-diabetes and no diabetes were more likely to be unemployed 89% (diabetes) versus 84% (pre-diabetes) and 73% (no diabetes) (p = 0.02) (Table 1). Patients with diabetes had a trend toward higher rates of substance abuse (37% for patients with diabetes versus 18% for patients with pre-diabetes and 23% for patients with no diabetes, *p* = 0.05). With regards to medical co-morbidities, patients with diabetes were almost twice as likely to have hypertension (22 versus 14 and 6%, *p* = 0.002), hyperlipidemia (28 versus 10 and 6%, *p* ≤ 0.001), obesity (44 versus 23 and 17%, *p* = 0.001) compared to those with pre-diabetes and no diabetes, respectively (Table 1). However, in a number of inpatients, there was no record of weight (*n* = 125, 37%), and in 34% (*n* = 115), there was no record of lipid profile (Table 1).

The most common psychiatric diagnoses were schizophrenia, depression, and substance abuse across the three groups. A high percentage of patients in all the three groups had two or more psychiatric diagnosis or a diagnosis that was not classified in those broad categories (Table 2).

**Table 2:** **The results of the multivariable logistic regression model investigating the association between independent variables with presence of diabetes compared to patients with pre-diabetes and no diabetes**.

	Diabetes versus pre-diabetes and no diabetes		
Clinical characteristics	Odds ratio	95% Confidence interval	*p*-Value
Age	1.04	1.01–1.07	0.02
Schizoaffective disorder	0.18	0.02–1.36	0.1
Substance abuse	2.13	1.03–4.43	0.04
Aripiprazole use	4.4	1.51–12.83	0.01
Hypertension	1.26	0.46–3.45	0.65
Hyperlipidemia	2.88	1.14–7.26	0.03
Obesity	1.96	0.90–4.26	0.09
Employed	0.57	0.20–1.59	0.28

*All p values were calculated from two-tailed tests of statistical significance and p < 0.05 was regarded as statistically significant*.

No statistically significant differences in duration of psychiatric illness between the groups were identified between the three groups [diabetes group, median (IQR): 12 years (6–18), pre-diabetes group: 10 years (4–19) versus no diabetes group: 9 years (3–18) (p = 0.215)] (Table 1).

**Figure 2:**
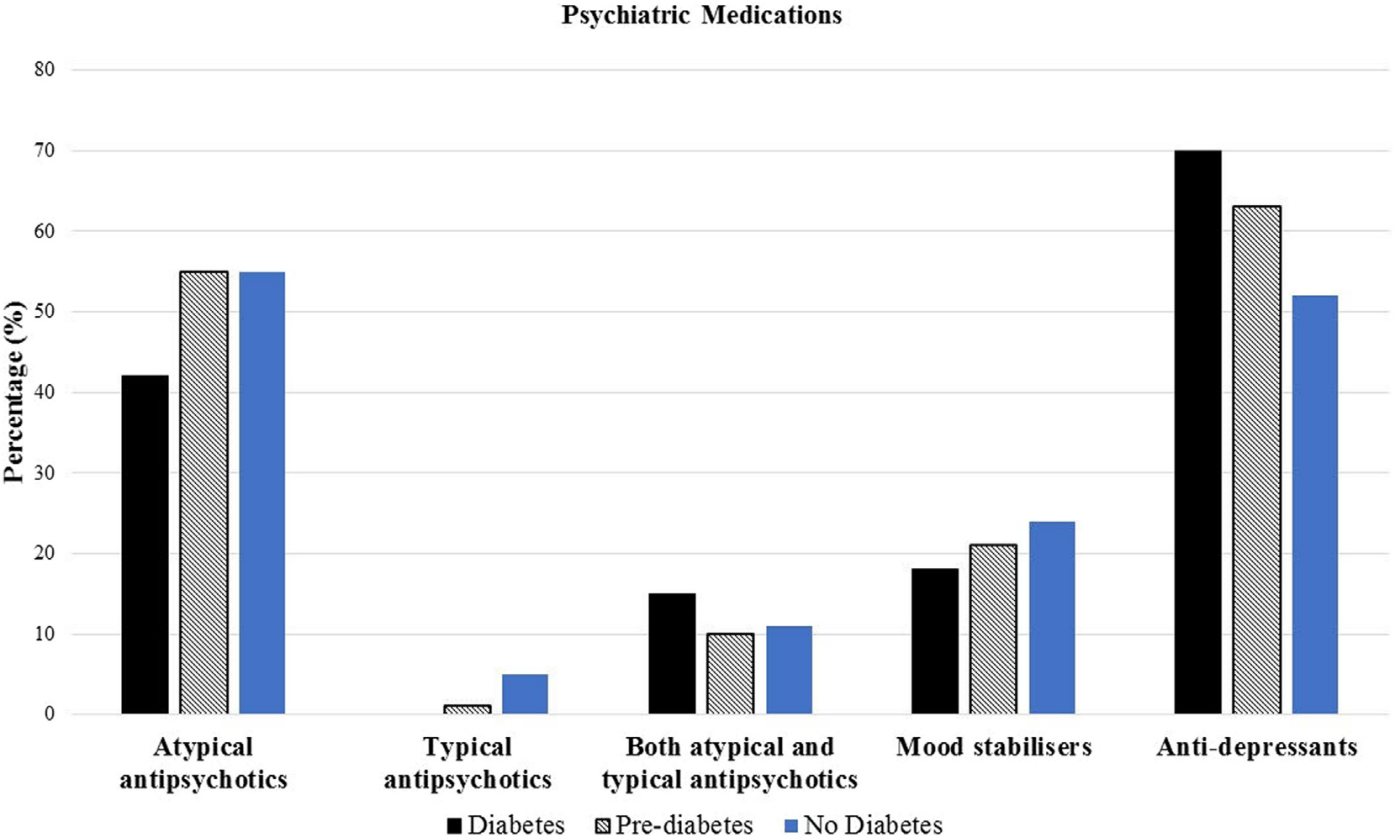
**Prevalence of baseline psychiatric medications among diabetes, pre-diabetes, and no diabetes groups**.

Atypical antipsychotic medication use was similar across each of the three groups (42% in diabetes, 55% in pre-diabetes, and 55% in no diabetes, *p* = 0.3) (Figure 2). No significant differences in the use of the different atypical antipsychotics between the diabetes, pre-diabetes, and no diabetes groups (0% in the diabetes group, 1% in the pre-diabetes group, and 5% in no diabetes group, *p* = 0.2) were detected. No significant differences in the use of both typical and atypical antipsychotics between the diabetes, pre-diabetes, and no diabetes groups (15% in the diabetes group, 10% in the pre-diabetes group, and 11% in the no diabetes group, *p* = 0.4) were detected. No significant differences in the use of mood stabilizers across the three groups (18% in the diabetes group, 21% in the pre-diabetes group, and 24% in the no diabetes group, *p* = 0.2). No significant differences in the use of anti-depressants across the three groups (70% in the diabetes group, 63% in the pre-diabetes group, and 52% in the no diabetes group, *p* = 0.1) (Figure 2).

### Univariate Analysis of Baseline Characteristics and Glycemic Status

In order to highlight risk factors associated with the presence of diabetes, for the remaining analyses, we pooled patients with pre-diabetes or no diabetes (*n* = 289, 86%) and compared to patients with diabetes (*n* = 46, 14%). Univariate analysis demonstrated that patients with diabetes were more likely to have a past history of substance abuse (37 versus 21%, *p* = 0.02), have a history of hypertension (22 versus 14 and 6%, *p* = 0.007), hyperlipidemia (28 versus 10 and 7%, *p* < 0.001), and obesity (44 versus 23 and 17%, *p* = 0.012), and have raised gamma-glutamyltransferase (43% versus 28%, p = 0.0009) compared to those with pre- diabetes or no diabetes. Patients with diabetes were more likely to be older [diabetes: median age 47, IQR (37–58) versus pre-diabetes and no diabetes (median age 40), IQR (34–48)] and less likely to be employed (diabetes: 11% versus pre-diabetes and without diabetes: 25%, *p* = 0.02). Patients with diabetes were also more likely to be on the atypical antipsychotic aripiprazole (15 versus 5%, *p* = 0.02) compared to those with pre-diabetes and no diabetes. Compared to patients with diabetes, patients with pre-diabetes and no diabetes were more likely to have schizoaffective disorder (12 versus 2%, *p* = 0.027) and other psychosis (10 versus 0%, *p* = 0.01).

### Multivariable Analysis

Multivariable logistic regression model investigating the associations with presence of diabetes included age, diagnosis of schizoaffective disorder, past history of substance abuse, presence of hypertension, dyslipidaemia, obesity, use of aripiprazole, and employment status as independent variables. On this adjusted analysis (Mean VIF = 1.12, Condition Number = 11.4), greater age (OR per year 1.04, 95% CI: 1.01–1.07, *p* = 0.02), past history of substance abuse (OR 2.13, 95% CI: 1.03–4.43, *p* = 0.04), presence of dyslipidaemia (OR 2.88, 95% CI: 1.14–7.26, *p* = 0.03) and use of aripiprazole (OR 4.4, 95% CI: 1.51–12.83, *p* = 0.01), were all statistically significantly associated with the presence of diabetes (Table 2).

### Length of Stay

The length of inpatient stay was longer in inpatients with diabetes when compared to pre-diabetes and no diabetes [median (IQR): 29 days (14–81) in diabetes group versus 18 days (9–29) in the pre-diabetes and no diabetes group (*p* = 0.0006)]. In inpatients whose LOS was less than 400 days (this LOS chosen to exclude outliers), a multivariable negative binomial regression analysis was undertaken. There were 11 outliers (3% of total patients) with LOS above 417 days. Based on this analysis, a psychiatric patient with diabetes was expected to stay in hospital 70% longer (Incidence rate ratio, 1.7 95% CI: 1.2–2.3, *p* = 0.001) than a patient with pre-diabetes or no diabetes with the similar status on all the different psychiatric diagnosis including schizophrenia, schizoaffective disorder, bipolar affective disorder, depression, other psychosis, substance abuse, and eating disorders.

To further investigate robustness of these findings, we undertook a similar analysis to determine the associations between diabetes status and LOS in patients who had admission for less than 90 days. Under this cutoff 30 patients were excluded (9% of the total number of patients). The results of this analysis were qualitatively similar. In those who had a LOS below 90 days, the multivariable negative binomial regression analysis model estimated that a psychiatric patient with diabetes trended toward a 30% longer LOS (Incidence Rate Ratio 1.3, 95% CI: 0.98–1.7; *p* = 0.07) than a patient with pre-diabetes or no diabetes with the similar status on all the different psychiatric diagnosis including schizophrenia, schizoaffective disorder, bipolar affective disorder, depression, other psychosis, substance abuse, and eating disorders.

## Discussion

First, this study demonstrated high prevalence of pre-diabetes and diabetes in the inpatient psychiatry population, compared to age-matched rates of prevalence of diabetes in the general community. Second, diabetes was associated with a more prolonged psychiatric inpatient LOS. Third, increasing age and substance abuse were associated with higher rates of diabetes in the inpatient psychiatry population, independent of other risk factors. Fourth, diabetes was associated with lower employment rates. Fifth, in one-third of inpatients, weight and lipid profile was not recorded.

The rates of pre-diabetes and diabetes in this study were 19% and 14% respectively. Prior studies have similarly reported high rates of diabetes in those patients with psychosis living in the community (6). In the current study, we demonstrate a snapshot of the glycemic status of the inpatient psychiatry population who are likely a more vulnerable group, with regards to higher psychiatric illness severity, chronic illness and disability.

In the current study, a longer duration of psychiatric illness was not associated with diabetes presence. A study of two epidemiological databases of psychiatric inpatients over two different time periods did not find any association between diabetes and serious mental illness but reported an association between diabetes and psychotropic treatment (15). The lack of association may be due to the small population size, the variety of mental health diagnosis, the short follow-up, and not accounting for confounding factors (15).

There was a significant gender bias skewed toward males, representing approximately 70% of psychiatric inpatients. This finding is in line with previous studies demonstrating that males present with psychiatric disorders requiring admission (16).

The most prevalent psychiatry diagnosis in our patient cohort included schizophrenia, depression, and substance abuse, which are reflective of the disease burden in the acute inpatient psychiatry patient. Substance abuse has been described in previous studies to be associated with the development of diabetes through multiple mechanisms, including hyperglycemia, hyperinsulinemia, poor overall physical health, higher rates of psychological distress, poor compliance to psychotropic therapy, and increasing rate of mental health admissions (6, 7, 17). It is, therefore, challenging to pinpoint one factor alone in the development of diabetes.

Many studies have shown that use of atypical psychotropic therapy has been associated with weight gain, impaired glucose metabolism, and increased risk of metabolic syndrome (18). Contrary to previous studies, we did not find a significant association between the type of psychotropic therapy patients received and prevalence of diabetes except in those inpatients who were taking aripiprazole. In fact aripiprazole has been shown in two studies to relate to a favorable metabolic profile (19, 20). Perhaps our data highlighted those individuals who were in the process of being transitioned to aripiprazole from a different psychotropic agent and hence this association may reflect the severity of underlying psychiatric disease and longer prior duration of antipsychotic use.

Limitations of the current study include lack of long-term medication exposures, including drug dosage prior to admission. Lifestyle factors, such as smoking, alcohol consumption, and substance abuse, may be underreported or inaccurately recorded. Clinical variables, such as weight and lipid profile, were missing in a significant proportion of the inpatients, perhaps reflecting the difficulty of obtaining a thorough physical examination and full metabolic profile in vulnerable psychiatry inpatients as has been described in previous studies (21, 22). Due to the limited data on the clinical characteristics, we were not able to make a comment on the presence of the metabolic syndrome in the current cohort. We were, therefore, not able to incorporate HbA1c into the revised definition of metabolic syndrome to enhance the detection of hyperglycemia (23). Additionally, ethnicity data was not routinely recorded.

Multivariable analysis highlighted variables associated with the presence of diabetes which may be useful in early flagging of those patients at highest risk of developing diabetes, namely, age, substance abuse, and presence of cardiovascular risk factors. Multivariable analysis also highlighted that patients with diabetes were more likely to have a longer length of inpatient psychiatry stay, suggesting perhaps that mental illness is more severe in people with diabetes.

However, given that this is an observational study, it is difficult to ascertain cause and effect. For example, the use of aripiprazole could reflect severity of mental illness and may be a confounder.

Current recommendations from Diabetes Australia are that individuals should be screened every 3 years from the age of 40 (24). The only relevant reference to screening those with mental illness is that the guideline suggests screening inpatients on antipsychotic medication alone, not including other psychiatric medications. A consensus statement developed by the ADA in 2004 discusses the screening for hyperglycemia in people treated specifically with antipsychotic medications (13). However, this may not capture other patients with mental illness who are on other psychiatric medications and may be at risk of developing diabetes. The New South Wales Health Clinical algorithm “Don’t Just Screen, Intervene” (25) recommends measurement of fasting glucose or HbA1c every 3–6 months in people receiving antipsychotic medications, for early detection and intervention. The National Institute for Health and Care Excellence Guidelines recommend diabetes screening prior to commencement of antipsychotic medication, 3 months later, then annually (26). Over one-third of our patient group did not have a weight or lipid profile recorded at baseline. This is a significant proportion of patients who are not getting optimal screening and, therefore, not being provided with the opportunity for optimization of management. Our current hospital guidelines suggest screening for diabetes as well other metabolic parameters in any patient prescribed psychotropic medications regardless of age (27). However, the challenge is obviously in implementing these guidelines as well ongoing management of these patients due to their potential lack of insight and care secondary to their mental illness. Barriers to screening, diagnosis, and monitoring of physical conditions in patients with mental illness are manifold and have already been described in many studies (28–31). Nevertheless, a hospital admission is an opportunity to screen for and initiate treatment of diabetes in all people with severe mental illness. Furthermore, it is an ideal opportunity to optimize therapy in a setting where admissions are often protracted and education in diabetes self-care and diet can be initiated once the patients is sufficiently well.

Metformin coupled with a lifestyle intervention program has been proposed in people at high risk of diabetes for cardio-metabolic protection as it has been shown to improve weight and metabolic parameters in normo-glycemic patients with psychosis commencing or receiving antipsychotic medication (32–34). Novel diabetic agents such as GLP-1 agonist are also being studied for management of diabetes and obesity in psychiatry populations on antipsychotic medications (35).

## Conclusion

The prevalence of type 2 diabetes and pre-diabetes in our inpatient psychiatry population was high compared to age-matched rates of prevalence of diabetes in the general community and reflected the poor physical health of patients with mental illness. There was a strong association between increased age, lower employment rates, and substance abuse with the prevalence of diabetes, independent of other risk factors, as well prolonged inpatient LOS. While there are good guidelines for diagnosis and managing diabetes in these patients, one-third of our patients did not have appropriate cardio-metabolic screening as per our current guidelines. The challenges lie in health professionals implementing these guidelines due to a number of factors, including well-defined disparities in the delivery of health care to people with severe mental illness and bidirectional lack of engagement. The management of glycemic status in psychiatric inpatients following discharge may also be challenging and hence routine HbA1c testing as an inpatient presents an opportunity to address and improve glycemic management during the inpatient stay.

## Author Contributions

PN and EIE initiated the study, participated in its design, conception, and co-ordination, helped in the collection of data for analysis and interpretation, drafting and editing of the article, and final approval for publication. LC contributed to the conception and design of the study, performed the statistical analysis, drafting and editing of the article, and final approval for publication. AK, HW, AM, AY, EC, and QL contributed to the design of the study, collection of data for analysis and interpretation, editing of the article, and final approval for publication. RK contributed to design of the study, analysis of data and drafting and editing of the article, and final approval for publication. MH contributed to the analysis of the data, interpretation of the data, editing of the article, and final approval for publication. GH, EO-J, RR, and KS contributed to the design of the study, analysis of data and editing of the article, and final approval for publication. JZ contributed to the design of the study, analysis of data, and editing of the article. All authors have read and approved the final version of the manuscript and final approval for publication. PN, LC, and EIE are the guarantors of this work and, as such, had full access to all the data in the study and take full responsibility for the integrity of the data and the accuracy of the data analysis and final approval for publication.

## Conflict of Interest Statement

The authors declare that the research was conducted in the absence of any commercial or financial relationships that could be construed as a potential conflict of interest. The handling editor declared a shared affiliation, though no other collaboration, with one of the authors (KS) and states that the process nevertheless met the standards of a fair and objective review.
